# Seeing spots: quantifying mother-offspring similarity and assessing fitness consequences of coat pattern traits in a wild population of giraffes (*Giraffa camelopardalis*)

**DOI:** 10.7717/peerj.5690

**Published:** 2018-10-02

**Authors:** Derek E. Lee, Douglas R. Cavener, Monica L. Bond

**Affiliations:** 1 Wild Nature Institute, Concord, NH, United States of America; 2 Department of Biology, Pennsylvania State University, University Park, United States of America; 3 Department of Evolutionary Biology and Environmental Studies, University of Zürich, Zürich, Switzerland

**Keywords:** Adaptation, Biometrics, Phenomics, Heritability, Natural selection, Remote measurement, Phenotypic selection, Coat pattern, Quantitative genetics, Visual animal biometry

## Abstract

Polymorphic phenotypes of mammalian coat coloration have been important to the study of genetics and evolution, but less is known about the inheritance and fitness consequences of individual variation in complex coat pattern traits such as spots and stripes. Giraffe coat markings are highly complex and variable and it has been hypothesized that variation in coat patterns most likely affects fitness by camouflaging neonates against visually hunting predators. We quantified complex coat pattern traits of wild Masai giraffes using image analysis software, determined the similarity of spot pattern traits between mother and offspring, and assessed whether variation in spot pattern traits was related to fitness as measured by juvenile survival. The methods we described could comprise a framework for objective quantification of complex mammal coat pattern traits based on photographic coat pattern data. We demonstrated that some characteristics of giraffe coat spot shape were likely to be heritable, as measured by mother-offspring regression. We found significant variation in juvenile survival among phenotypic groups of neonates defined by multivariate clustering based on spot trait measurement variables. We also found significant variation in neonatal survival associated with spot size and shape covariates. Larger spots (smaller number of spots) and irregularly shaped or rounder spots (smaller aspect ratio) were correlated with increased survival. These findings will inform investigations into developmental and genetic architecture of complex mammal coat patterns and their adaptive value.

## Introduction

Complex color patterns such as spots and stripes are found on many animal species and these phenotypic traits are hypothesized to play adaptive roles in predator and parasite evasion, thermoregulation, and communication (Cott, 1940; Caro, 2005). Many foundational studies of coloration using starkly different color morphs from diverse taxa such as insects (Kettlewell, 1955; Wittkopp et al., 2003), mice (Morse, 1978; Russell, 1985; Bennett & Lamoreux, 2003), reptiles (Rosenblum, Hoekstra & Nachman, 2004; Calsbeek, Bonneaud & Smith, 2008), fish (Endler, 1983; Irion, Singh & Nuesslein-Volhard, 2016), and birds (Roulin, 2004) demonstrated Mendelian inheritance and natural selection, and discovered genes that cause color morph mutations (Hoekstra, 2006; Protas & Patel, 2008; San-Jose & Roulin, 2017). Individual variation in a complex color pattern trait of spot size was also part of the earliest work on genetics and inheritance (Wright, 1917). Measuring individual variation in complex color patterns, especially detailed measurements such as animal biometrics (Kühl & Burghardt, 2013), can provide novel insight into developmental and genetic architecture (Bowen & Dawson, 1977; Klingenberg, 2010; San-Jose & Roulin, 2017), and the adaptive value of the patterns (Hoekstra, 2006; Allen et al., 2011), as well as benefitting studies of behavior (Lorenz, 1937; Whitehead, 1990), population biology (Holmberg, Norman & Arzoumanian, 2009; Lee & Bolger, 2017), and the growing field of phenomics (Houle, Govindaraju & Omholt, 2010). A few methods to robustly quantify continuous variation among individuals in complex color patterns have been developed for general use (Schneider, Rasband & Eliceiri, 2012; Van Belleghem et al., 2018) and specific taxa such as fishes (Endler, 1980; Holmberg, Norman & Arzoumanian, 2009), butterflies (Le Poul et al., 2014), penguins (Sherley et al., 2010), and primates (Allen, Higham & Allen, 2015). We see a need for more tools and techniques to reliably quantify individual variation in complex coat pattern traits in wild populations (Eizirik et al., 2010; Willisch, Marreros & Neuhaus, 2013), and studies that use quantitative genetics and demographic methods to investigate heritability and adaptive significance of those traits in wild mammal populations (Kruuk, Slate & Wilson, 2008; Kaelin et al., 2012).

**Figure 1:**
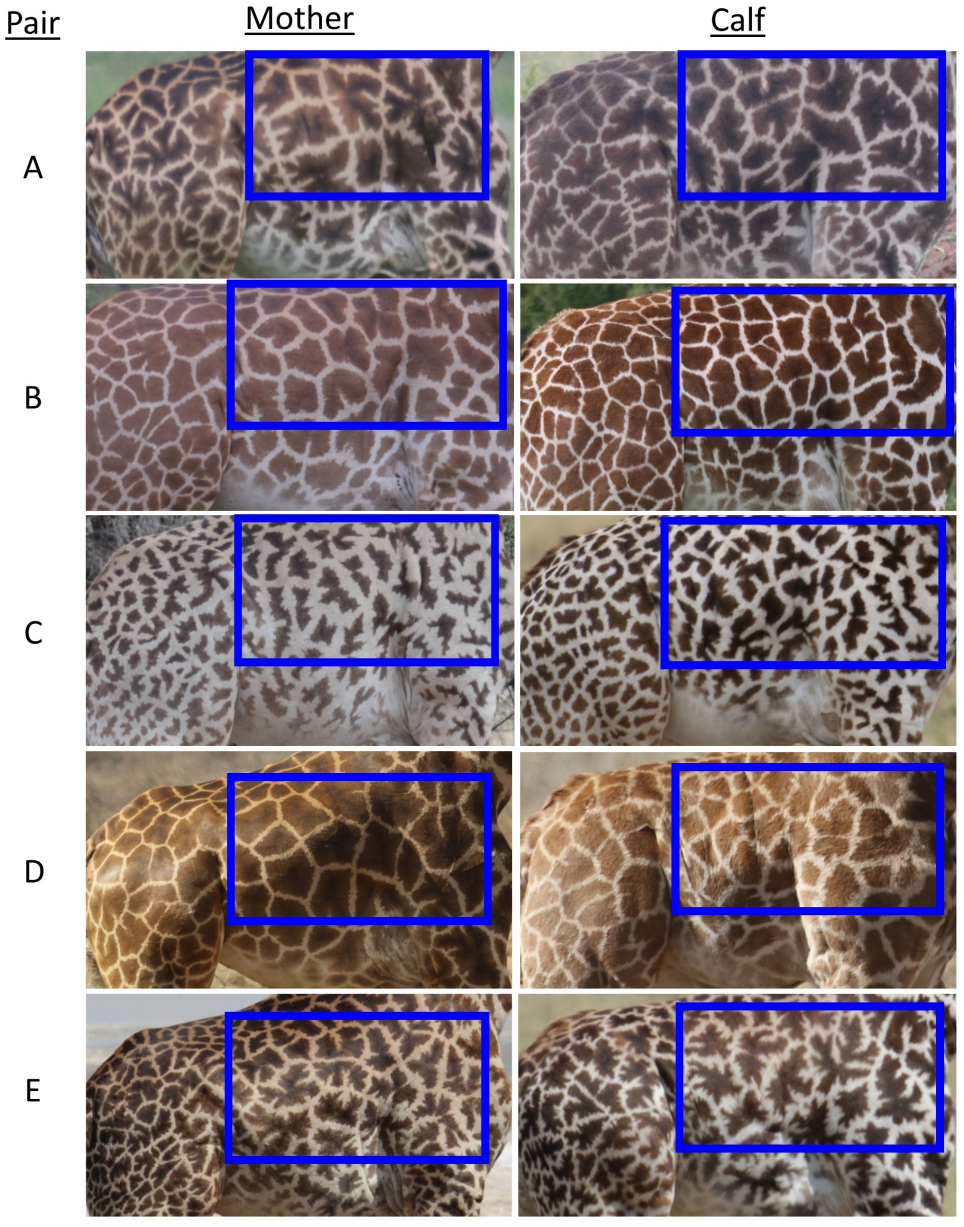
Representative images of spot patterns of mother-calf pairs of Masai giraffes (*Giraffa camelopardalis tippelskirchii*) from the Tarangire ecosystem, Tanzania used in this study. The blue rectangle shows the area analysed using ImageJ to characterize spot pattern traits. All photos by DE Lee. (A) Mother-calf pair number 1, (B) mother-calf pair number 2, (C) mother-calf pair number 3, (D) mother-calf pair number 4.

The coat patterns of Masai giraffes (*Giraffa camelopardalis tippelskirchii*) are complex and show a high degree of individual variation (Dagg, 1968; Fig. 1). Masai giraffes’ spots vary in color and shape from those that are nearly round with very smooth edges (low tortuousness), to extremely elliptical with incised or lobate edges (high tortuousness). Giraffe skin pigmentation is uniformly dark grey (Dimond & Montagna, 1976), but the spots that make up their coat markings are highly variable in traits such as color, roundness, and perimeter tortuousness. This variation has been used to classify subspecies (Lydekker, 1904), and to reliably identify individuals because patterns do not change with age (Foster, 1966; Bolger et al., 2012; Dagg, 2014). Dagg (1968) first presented evidence from a small zoo population that the shape, number, area, and color of spots in giraffe coat patterns may be heritable, but analysis of spot traits in wild giraffes, and objective measurements of spot characteristics in general have been lacking.

It has been hypothesized that giraffe coat patterns evolved to camouflage neonates whose primary defense against predation is concealment (Langman, 1977; Mitchell & Skinner, 2003), thus the most likely fitness effects from variation in coat patterns should be variation in juvenile survival. Giraffe calves spend much of their time, day and night, hiding in the dappled light of trees and bushes and their ability to match this background should influence detection by visually hunting predators such as lions and hyenas (Endler, 1978; Merilaita, Scott-Samuel & Cuthill, 2017). Background matching, the adaptation of an animal’s coloration to mimic its average background and reduce detection by visually hunting predators, is a common form of camouflage (Endler, 1978; Merilaita, Scott-Samuel & Cuthill, 2017). Alternative hypotheses about the adaptive value of giraffe coat markings include thermoregulation (Skinner & Smithers, 1990), and in this social species with good visual sensory perception (Dagg, 2014; VanderWaal et al., 2014), markings could also facilitate individual recognition (Tibbetts & Dale, 2007) and kin recognition (Beecher, 1982; Tang-Martinez, 2001). To date, no evidence has been presented for any of these hypotheses.

Our purpose in this study was to: (1) demonstrate the use of public domain image analysis software ImageJ (Schneider, Rasband & Eliceiri, 2012) to extract patterns from image data and quantify multiple aspects of the complex coat patterns of wild Masai giraffes; (2) use quantitative genetics methods (parent–offspring regression) to quantify the proportion of observed phenotypic variation of a trait that is shared between mother and offspring; and (3) determine whether variation in complex coat pattern traits was related to a measure of fitness (survival) and thereby infer the effect of natural selection (viability selection) on giraffe coat patterns (Lande & Arnold, 1983; Falconer & Mackay, 1996).

## Materials & Methods

As a general overview, our methods were to: (1) collect field data in one area of Tanzania as digital images of giraffes to be used for spot pattern and survival analyses; (2) extract patterns from images; (3) quantify giraffe patterns by measuring 11 spot traits; (4) use principal components analysis (PCA) to reduce the dimensionality of the spot traits; (5) use mother-offspring regressions to estimate the phenotypic similarity between mother and offspring of the 11 spot traits and the 1st two dimensions of the PCA; (6) use k-means clustering to assign giraffe calves into phenotypic groups according to their spot pattern traits; (7) use capture-mark-recapture analysis to estimate survival and determine whether there are fitness differences among the phenotypic groups; (8) use capture-mark-recapture analysis to determine whether there are fitness effects from any particular spot traits.

This research was carried out with permission from the Tanzania Commission for Science and Technology (COSTECH), Tanzania National Parks (TANAPA), the Tanzania Wildlife Research Institute (TAWIRI), African Wildlife Foundation, and Manyara Ranch Conservancy.

### Field Data Collection

This study used data from individually identified, wild, free-ranging Masai giraffes in a 1,700 km^2^ sampled area within a 4,400 km^2^ region of the Tarangire Ecosystem, northern Tanzania, East Africa. Data were collected as previously described in Lee et al. (2016a). We collected data during systematic road transect sampling for photographic capture-mark-recapture (PCMR). We conducted 26 daytime surveys for giraffe PCMR data between January 2012 and February 2016. We sampled giraffes three times per year around 1 February, 1 June, and 1 October near the end of every precipitation season (short rains, long rains, and dry, respectively) by driving a network of fixed-route transects on single-lane dirt tracks in the study area. We surveyed according to Pollock’s robust design sampling framework (Pollock, 1982; Kendall, Pollock & Brownie, 1995), with three occasions per year. Each sampling occasion was composed of two sampling events during which we surveyed all transects in the study area with only a few days interval between events. Each sampling occasion was separated by a 4-month interval (4.3 years × 3 occasions year^−1^ × 2 events occasion^−1^ = 26 survey events).

During PCMR sampling events, a sample of individuals were encountered and either ‘sighted’ or ‘resighted’ by slowly approaching and photographing the animal’s right side from approximately 150 m at a perpendicular angle (Canon 40D and Rebel T2i cameras with Canon Ultrasonic IS 100–400 mm lens; Canon USA, Inc., One Canon Park, Melville, New York, USA). We identified individual giraffes using their unique and unchanging coat patterns (Foster, 1966; Dagg, 2014) with the aid of pattern-recognition software Wild-ID (Bolger et al., 2012). We attempted to photograph every giraffe encountered, and recorded sex and age class based on physical characteristics. We assigned giraffes to one of four age classes for each observation based on the species’ life history characteristics and our sampling design: neonate calf (0–3 months old), older calf (4–11 months old), subadult (1–3 years old for females, 1 –6 years old for males), or adult (>3 years for females, >6 years for males) using a suite of physical characteristics (Strauss et al., 2015), and size measured with photogrammetry (Lee et al., 2016a). In this analysis, we used only adult females and animals first sighted as neonate calves.

All animal work was conducted according to relevant national and international guidelines. This research was carried out with permission from the Tanzania Commission for Science and Technology (COSTECH) Research Permit numbers 2017-163-ER-90-172, 2016-146-ER-2001-31, 2015-22-ER-90-172, 2014-53-ER-90-172, 2013-103-ER-90-172, 2012-175-ER-90-172, 2011-106-NA-90-172, Tanzania National Parks (TANAPA), the Tanzania Wildlife Research Institute (TAWIRI). No Institutional Animal Care and Use Committee (IACUC) approval was necessary because animal subjects were observed without disturbance or physical contact of any kind.

### Quantification of spot patterns

We extracted patterns and analysed spot traits of each animal within the shoulder and rib area by cropping all images to an analysis rectangle that fit horizontally between the anterior edge of the rear leg and the chest, and vertically between the back and where the skin folded beneath the posterior edge of the foreleg (Fig. 1). For color trait analysis, we used the Color Histogram procedure of ImageJ (Schneider, Rasband & Eliceiri, 2012) full-color images of the analysis rectangle. We extracted coat patterns using ImageJ to convert full-color images of the analysis rectangle to 8-bit greyscale images, then converted to bicolor (black and white) using the Enhance Contrast and Threshold commands (Schneider, Rasband & Eliceiri, 2012). We quantified 10 spot trait measurements of each animal’s extracted coat pattern using the Analyze Particles command in ImageJ (Schneider, Rasband & Eliceiri, 2012). To account for differences in image resolution and animal size (including age-related growth), and to obtain approximately scale-invariant standard images of each animal, we set the measurement unit of each image equal to the number of pixels in the height of the analysis rectangle. Therefore all measurements are in giraffe units (GU), where 1 GU = height of the analysis rectangle (Fig. 1). We excluded spots cut off by the edge of the analysis rectangle to avoid the influence of incomplete spots, and we also excluded spots whose area was <0.00001 GU^2^ to eliminate the influence of speckles.

We characterized each animal’s coat spot pattern traits within the analysis rectangle using the following 11 metrics available in ImageJ (10 measurements plus color): number of spots; mean spot size (area); mean spot perimeter; mean angle between the primary axis of an ellipse fit over the spot and the *x*-axis of the image; mean circularity (4 *π* × [Area]/[Perimeter] ^2^ with a value of 1.0 indicating a perfect circle and smaller values indicating an increasingly elongated shape); mean maximum caliper (the longest distance between any two points along the spot boundary, also known as Feret diameter); mean Feret angle (the angle [0 to 180 degrees] of the maximum caliper); mean aspect ratio (of the spot’s fitted ellipse); mean roundness (4 × [Area]*π* × [Major axis]^2^ or the inverse of aspect ratio); mean solidity ([Area]/[Convex area], also called tortuousness); and mode shade ([65536 ×r] + [256 ×g] + [b] using RGB (red, green, blue) values from color histogram from full color photos). Circularity describes how close the spot is to a perfect circle, and is positively correlated with the trait of roundness. Solidity describes how smooth and entire the spot edges are versus tortuous, ruffled, lobed, or incised and is negatively correlated with the trait of perimeter. Number is negatively correlated with size and perimeter, with all three metrics indicating spot size. See Table S2 for all correlations among traits.

We quantified total phenotypic variation in spot trait values by reporting the mean, SD, and coefficient of variation (CV) of each trait. We also quantified the repeatability (R) as the within-individual correlation among measurements (Nakagawa & Schielzeth, 2010) of spot pattern trait measurement technique for the same animal made on different photos from different dates using a set of 30 animals with >2 images per animal using package rptR (Stoffel, Nakagawa & Schielzeth, 2017). We performed a principal components analysis (PCA; Hotelling, 1933) on the covariance matrix of the 10 spot trait measurements (standardized to *z*-scores) to examine the patterns of variation and covariation among the spot measurement data and to compute two summary dimensions explaining the 10 measurements (color was not included). We performed k-means clustering to divide animals into ‘coat pattern phenotypes,’ phenotypic groups based upon their spot trait characteristics (MacQueen, 1967; Hartigan, 1975). The optimal number of phenotypic groups was determined by the gap statistic (Tibshirani, Walther & Hastie, 2001). We performed statistical operations using R (R Core Development Team, 2017) packages lmer (Bates et al., 2015), FactoMineR (Le, Josse & Husson, 2008), and rptR (Stoffel, Nakagawa & Schielzeth, 2017).

### Mother-offspring similarity of spot traits

The (narrow sense) heritability of a trait (symbolized *h*^2^) is the proportion of its total phenotypic variance due to additive genetic effects, or available for selection to act upon. Parent-offspring (PO) regression is one of the traditional quantitative genetics tools used to test for heritable additive genetic variation (Falconer & Mackay, 1996). We used mother-offspring regression to compute similarity where heritability is 2 × the slope of the regression. PO regression studies cannot distinguish among phenotypic similarity due to genetic heritability, maternal effects, or shared environmental effects (Falconer & Mackay, 1996); it is, however, one of the few methods available when information on other kin relations is lacking. Pigmentation traits in mammals are known to have a strong genetic basis (Bennett & Lamoreux, 2003; Hoekstra, 2006), supporting the interpretation of PO regression as indicating a genetic component. We expect minimal non-random variation due to environmental effects because the calves were all born in the same area with the same vegetation communities during a relatively short time period of average climate and weather with no spatial segregation by coat pattern phenotype (Fig. S1). The animal model was not an improvement because we do not know fathers, and we had no known siblings in our dataset, therefore PO regression is the most appropriate tool for our estimates of heritability, with the caveat that there are potentially environmental and maternal effects also present.

We identified 31 mother-calf pairs by observing extended suckling behavior (>5 s). Wild female giraffes very rarely suckle a calf that is not their own (Pratt & Anderson, 1979). We examined all identification photographs for individuals in known mother-calf pairs, and selected the best-quality photograph for each animal based on focus, clarity, perpendicularity to the camera, and unobstructed view of the torso.

We predicted spot pattern traits of a calf would be correlated with those of its mother. We estimated the mother-offspring similarity for each of the 11 spot trait measurements, and the first two dimensions generated by the PCA. When we examined the 11 individual spot traits, we used the Bonferroni adjustment (*α*/number of tests) to account for multiple tests and set our adjusted *α* = 0.0045. We performed statistical operations in R (R Core Development Team, 2017). We tested that the PO regressions for each trait met assumptions of normality of residuals and homoscedasticity using qqPlot and ncvTest functions in package car in R (Fox & Weisberg, 2011).

### Associations between spot patterns and juvenile survival

We assembled encounter histories for 258 calves first observed as neonates for survival analysis. For each calf we selected the best-quality calf-age (age <  6 mo) photograph based on focus, clarity, perpendicularity to the camera, and unobstructed view of the torso, and ran the photographs through the ImageJ analysis to quantify each individual’s coat spot traits. We analysed survival using capture-mark-recapture apparent survival models that account for imperfect detectability during surveys (White & Burnham, 1999). No capture-mark-recapture analyses except ‘known fate’ models can discriminate between mortality and permanent emigration, therefore when we speak of survival it is technically ‘apparent survival,’ but during the first seasons of life we expected very few calves to emigrate from the study area, and if any did emigrate permanently this effect on apparent survival should be random relative to their spot pattern characteristics.

We ran two analyses of calf survival. In the first, we estimated age-specific seasonal (4-month seasons) survival (up to 3 years old) according to coat pattern phenotype groups with calves assigned to groups by k-means clustering of their overall spot traits. We compared five models, a null model of one group, age + three groups, age ×3 groups, age + four groups, and age × four groups, to examine whether coat pattern phenotypes affected survival differently at different ages. In the second survival analysis, we estimated survival as a function of individual covariates of specific spot traits including linear and quadratic relationships of all 11 spot traits and the first two PCA dimensions on juvenile survival to examine whether directional, disruptive, or stabilizing selection was occurring (Lande & Arnold, 1983; Falconer & Mackay, 1996). To determine at what age specific spot traits had the greatest effect of survival, we examined survival as a function of spot traits during 3 age periods: the first season of life, first year of life, and first three years of life.

We used Program MARK to analyse complete capture-mark-recapture encounter histories of giraffes first sighted as neonates (White & Burnham, 1999). We analysed our encounter histories using Pollock’s Robust Design models to estimate age-specific survival (Pollock, 1982; Kendall, Pollock & Brownie, 1995), and ranked models using AIC_c_ following Burnham & Anderson (2002). We used weights (W) and likelihood ratio tests as the metrics for the strength of evidence supporting a given model as the best description of the data (Burnham & Anderson, 2002). Due to model selection uncertainty in the analysis of phenotypic groups, we present model-averaged parameter values and based all inferences on these model-averaged values (Burnham & Anderson, 2002). We considered factors to be statistically significant if the 95% confidence interval of the beta coefficient did not include zero.

Based on previous analyses for this population (Lee et al., 2016a, Lee et al., 2016b), we constrained parameters for survival (S) and temporary emigration (*γ*′ and *γ*″) to be linear functions of age (symbolized ‘A’), and capture and recapture (c and p) were time dependent (symbolized ‘t’), so the full model was: {(S(A), *γ*′ (A), *γ*″ (A), c(t), p(t)}. Giraffe calf survival does not vary by sex (Lee et al., 2016b), so we analysed all calves together as an additional constraint on the number of parameters estimated. We tested goodness-of-fit in encounter history data using U-CARE (Choquet et al., 2009), and we found some evidence for lack of fit (${\chi }_{62}^{2}=97$, *P* = 0.01), but because the computed $\hat {c}$ adjustment was <3 ($\hat {c}=1.5$), we felt our models fit the data adequately and we did not apply a variance inflation factor (Burnham & Anderson, 2002; Choquet et al., 2009).

We have deposited the primary data underlying these analyses as follows: sampling locations, original data photos, and spot trait data: Dryad DOI: https://doi.org/10.5061/dryad.6514r.

## Results

**Figure 2:**
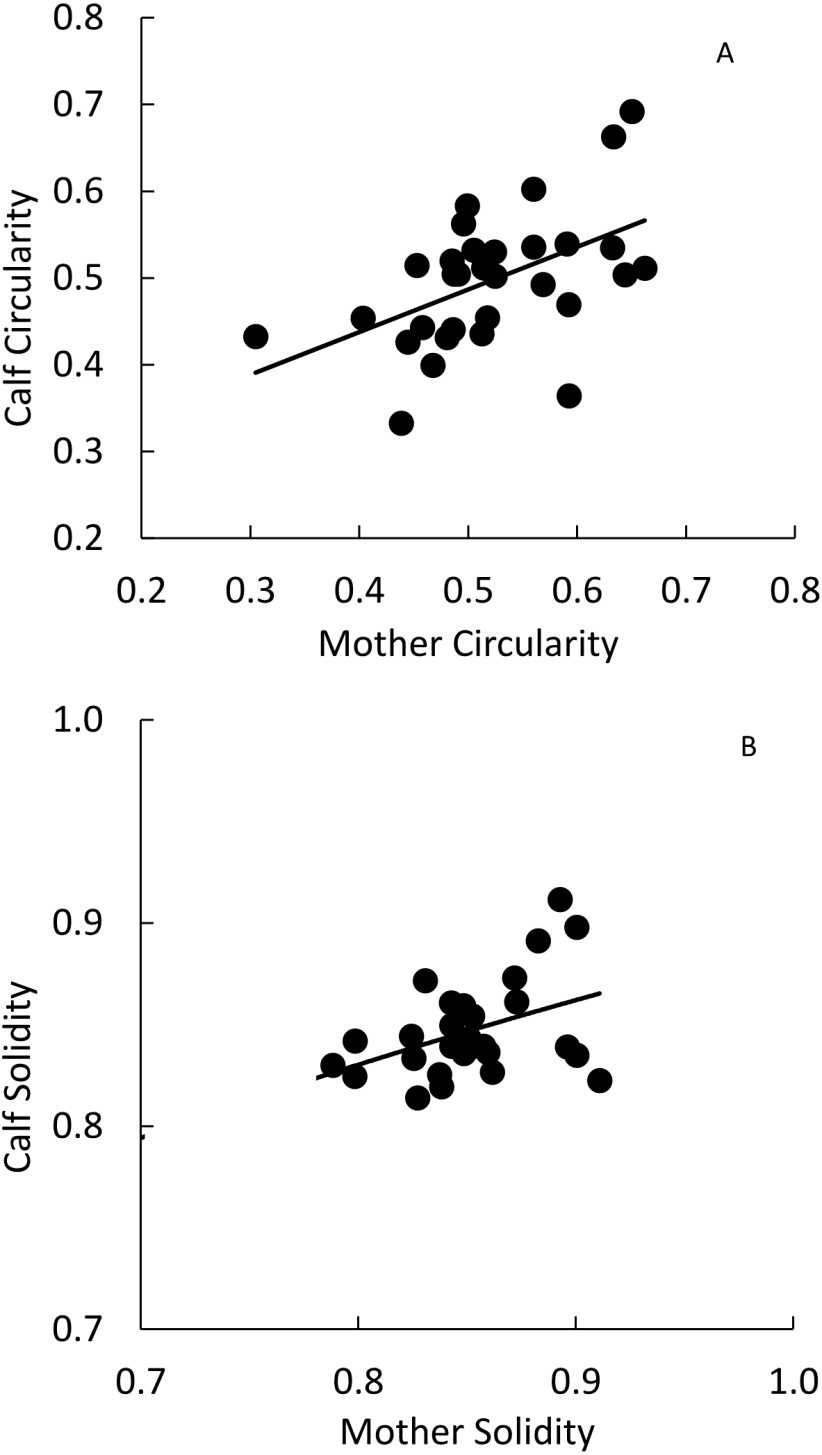
Mother-offspring regressions for (A) circularity and (B) solidity values of Masai giraffes in northern Tanzania. These shape traits were significantly correlated between mother and calf.

We were able to extract patterns and quantify 11 spot traits using ImageJ, and found measurements were highly repeatable with low variation in measurements from different photos of the same individual (Table 1). From our 31 mother-calf pairs, all PO regressions met assumptions of normality of residuals and homoscedasticity (Fig. S2). We found two spot shape traits, circularity and solidity (tortuousness) (Fig. S3) had significant PO slope coefficients between calves and their mothers indicating similarity (Table 1 and Fig. 2).

**Table 1:** Summary statistics for mother-offspring regressions of spot traits of Masai giraffes in northern Tanzania. Mean trait values, SD (standard deviation), CV (among-individuals coefficient of variation), Repeatability (within-individual correlation among measurements from different pictures of the same individual), Parent-offspring (PO) slope coefficients, *F*-statistics, and *P* values are provided. Statistically significant heritable traits are in bold.

	Number	Area	Perimeter	Angle	Circularity	Maximum caliper	Feret angle	Aspect ratio	Roundness	Solidity	Mode shade	PCA 1st dimension	PCA 2nd dimension
Mean	18.9	0.04	0.99	87.96	0.51	0.29	88.2	1.69	0.63	0.84	6924050		
SD	7.5	0.01	0.25	15.39	0.08	0.06	14.5	0.15	0.04	0.04	3930565		
CV	0.40	0.39	0.25	0.17	0.15	0.19	0.16	0.09	0.06	0.05	0.57		
Repeatability (R)	0.78	0.78	0.74	0.92	0.82	0.84	0.86	0.9	0.94	0.96	0.74		
SE of R	0.30	0.23	0.19	0.19	0.31	0.32	0.16	0.22	0.21	0.27	0.24		
*P* value (R)	0.003	0.002	0.002	0.001	0.008	0.009	0.002	0.001	0.001	0.002	0.002		
PO Slope Coefficient	0.20	0.20	0.27	0.04	**0.52**	0.21	−0.15	0.19	0.08	**0.53**	0.44	0.39	0.21
PO Coefficient SE	0.23	0.21	0.18	0.20	**0.16**	0.21	0.15	0.18	0.17	**0.17**	0.22	0.21	0.19
Heritability	0.40	0.40	0.54	0.08	**1.04**	0.42	0.30	0.38	0.16	**1.06**	0.88	0.78	0.42
F_1,29_	0.76	0.87	2.27	0.04	**9.97**	1.01	0.91	1.11	0.19	**9.73**	4.16	3.45	1.11
*P* value (PO)	0.39	0.36	0.14	0.84	**0.0037**	0.32	0.35	0.30	0.66	**0.0041**	0.05	0.07	0.30

**Figure 3:**
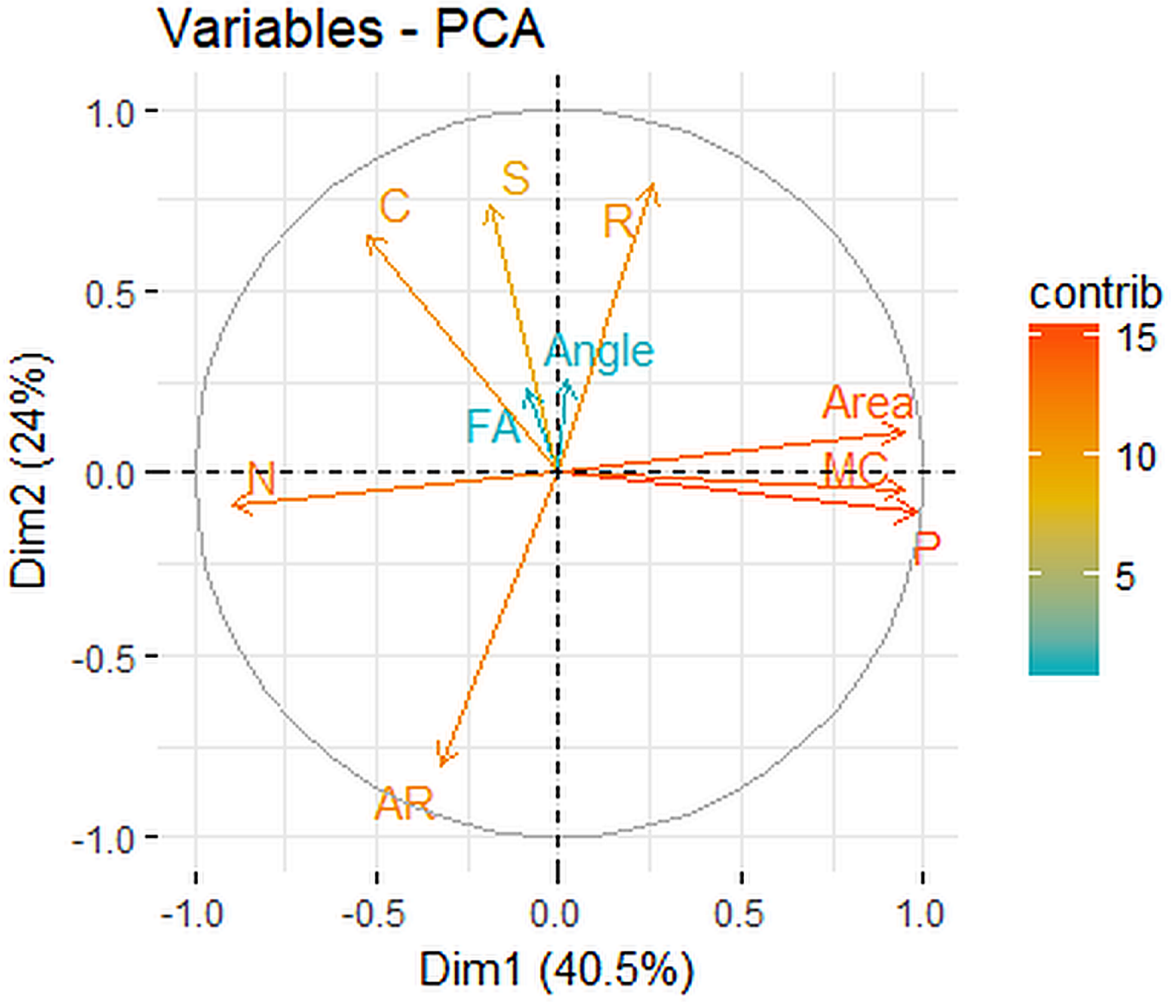
Contributions of 10 trait measurement variables to the first 2 dimensions of the principal components analysis of giraffe spots. The first dimension (Dim1) was composed primarily of spot size-related traits (perimeter, maximum caliper, area, and number of spots), the second dimension (Dim2) was composed primarily of spot shape traits (aspect ratio, roundness, solidity, and circularity). C, circularity, S, solidity, R, roundness, N, number of spots, AR, aspect ratio, MC, maximum caliper, P, perimeter.

The first dimension from the PCA (from 258 calves, including the 31 calves used to estimate heritability) was composed primarily of spot size-related traits (perimeter, maximum caliper, area, and number) such that increasing dimension 1 meant increasing spot size. Dimension 1 explained 40.5% of the variance in the data (Fig. 3). The second dimension was composed primarily of spot shape traits (aspect ratio, roundness, solidity, and circularity) such that increasing dimension 2 meant increasing roundness and circularity while decreasing dimension 2 meant more tortuous edges and irregular shapes. Dimension 2 explained 24.0% of the variation in the data (Fig. 3). The variance explained by additional dimensions and the contributions of variables to the first two dimensions are given in Table S1 and (Fig. S4). None of the dimensions from the PCA had significant PO regression slopes (Table 1). Correlations among variables are given in Table S2.

**Figure 4:**
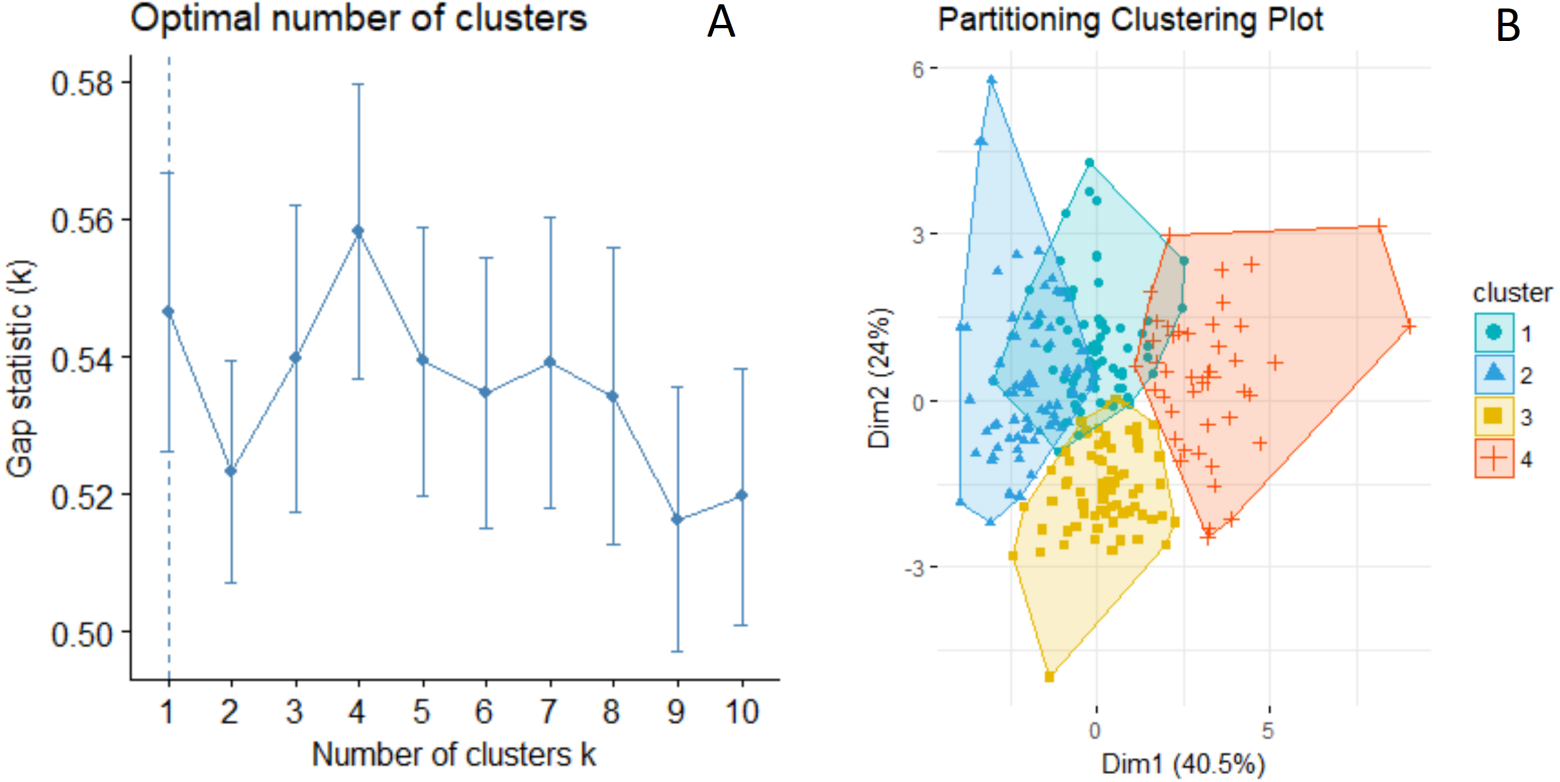
Results from k-means cluster analysis of giraffe spot patterns to define phenotypic groups. (A) Gap statistic for different numbers of groups. (B) Four clusters mapped in PCA space.

**Figure 5:**
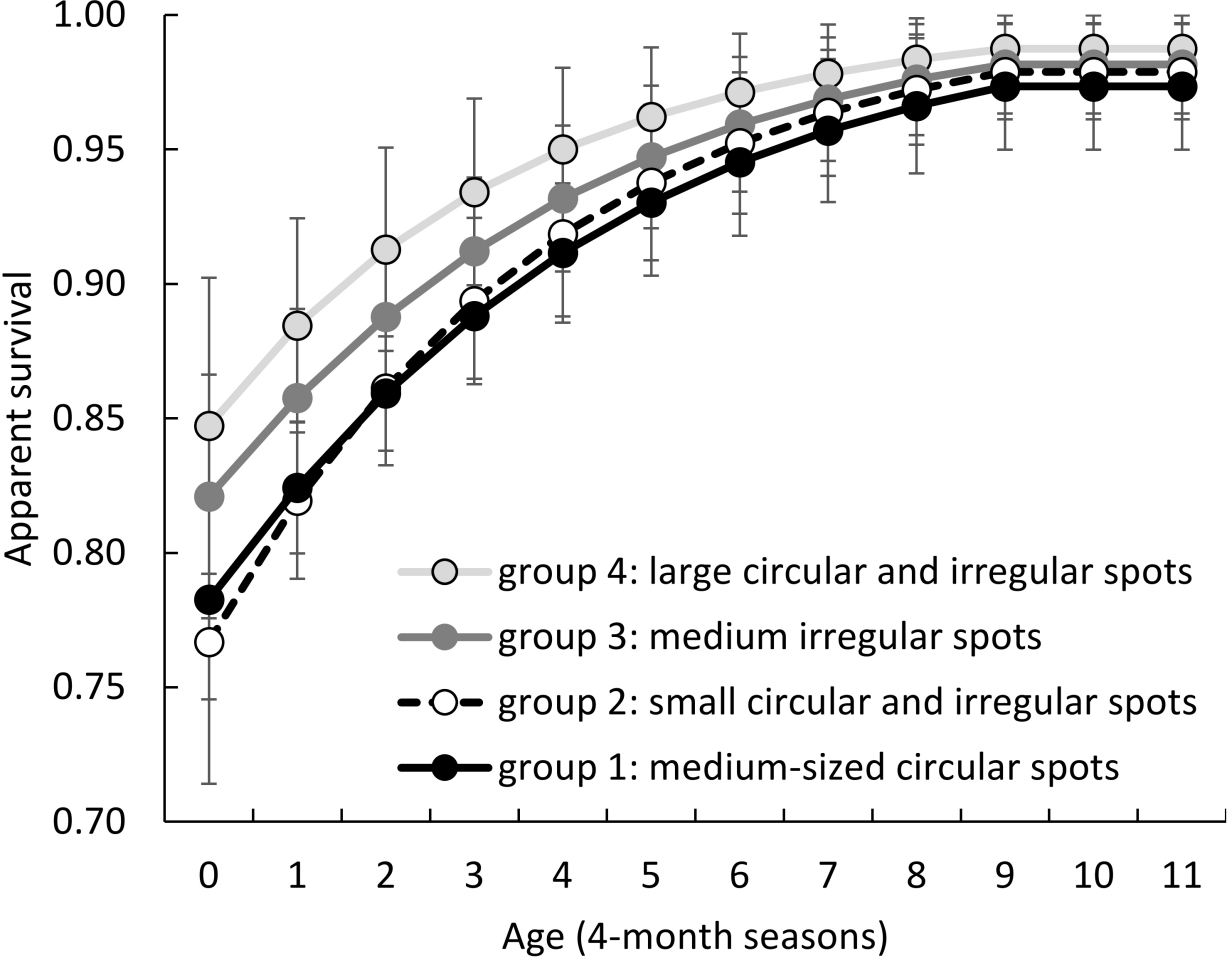
Model-averaged seasonal (4 months) apparent survival estimates for coat pattern phenotypic groups of giraffes defined by k-means clustering of their spot pattern traits. There was evidence for significant differences in survival among phenotypic groups during the younger ages, but those differences were greatly reduced as the animals approached adulthood (age 9–11 seasons). Error bars are ±1 SE.

Gap statistics indicated either one, three or four phenotypic groups was the optimal number of clusters for k-means clustering (Fig. 4). We examined survival differences among three and four phenotypic groups relative to a one-group (null) model. In the four-group definition, group 1 had medium-sized circular spots, group 2 had small-sized circular and irregular spots, group 3 had medium-sized irregular spots, and group 4 had large circular and irregular spots (Figs. 3 and 4). Groups 1 and 2 had a large amount of overlap in PCA variable space (Fig. 4), so we created three phenotypic groups by lumping the two overlapping groups. Our survival analysis of 258 calves divided into four phenotypic groups based on their spot traits indicated that the one-group model was top-ranked, but AIC_c_ weights showed there was some evidence for survival variation among the 4 phenotypic groups (Table 2). The 3 phenotypic group model found significant differences in survival according to group (Table 2, the 95% confidence interval of the beta coefficient did not include zero for lumped groups 1 and 2 =  − 0.717, 95% CI = −1.408 to −0.002). Model-averaged seasonal apparent survival estimates indicated differences in survival of 0.04 to 0.07 existed among phenotypic groups during the first season of life, but those differences were greatly reduced in ages 1 and 2 years old (Fig. 5).

**Table 2:** Model selection results for giraffe calf survival according to phenotypic groups defined by spot traits. Model weights indicated some evidence for phenotypic group effects on survival. Notation ‘A’ indicates a linear trend with age. Additive models indicate groups shared a common slope coefficient, but had different intercepts; multiplicative models indicated groups had different intercepts and different slopes. Minimum AIC_c_ = 3,236.38, *W* = AIC_c_ weight, *k* =  number of parameters.

Model	ΔAIC_c_	*W*	*k*
A + 3 groups	0	0.43	36
A + 1 group	0.94	0.27	34
A + 4 groups	2.06	0.15	37
A ×4 groups	3.01	0.09	40
A ×3 groups	3.91	0.06	38

**Figure 6:**
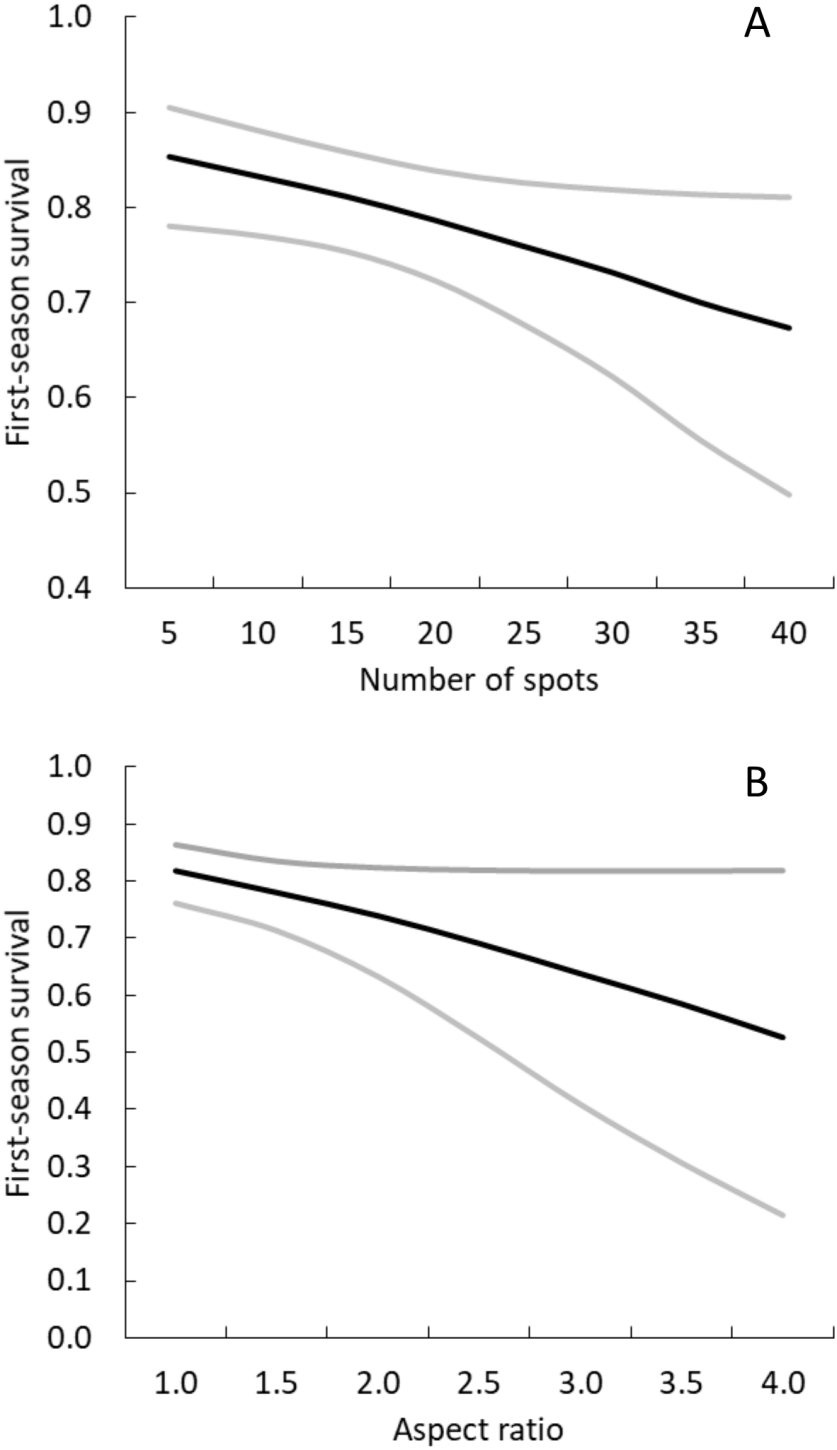
Survival of neonatal giraffes during their first 4 months of life was negatively correlated with (A) number of spots and (B) aspect ratio. Number of spots and aspect ratio are inversely related to spot size and roundness (the variables used when describing coat pattern phenotypic groups). Black lines are model estimates, grey lines are 95% confidence intervals.

We found two specific spot traits significantly affected survival during the first season of life (number of spots and aspect ratio; beta _number of spots_ =  − 0.031, 95% CI = −0.060 to −0.007; beta _aspect ratio_ =  − 0.466, 95% CI = −0.957 to −0.002). Both number of spots and aspect ratio were negatively correlated with survival during the first season of life (Fig.  6). No other trait during any age period significantly affected juvenile survival (all beta coefficient 95% CIs included zero), but model selection uncertainty was high (Table  3). Number of spots and aspect ratio were not correlated with each other (Table S2).

**Table 3:** Model selection results for giraffe calf survival as a linear or quadratic function of spot trait covariates during the first season (4 months), first year, and first 3 years of life. Confidence intervals of beta coefficients for two traits excluded zero (number of spots, and aspect ratio), indicating evidence for significant spot trait effects on calf survival during the first season of life. Model structure in all cases was {*S*(*A* + Covariate)*g*″(*A*)*g*′(*A*)*p*(*t*)*c*(*t*)} with covariate structure in survival. Notation ‘*A*’ indicates a linear trend with age, ‘*t*’ indicates time dependence. Minimum AIC_c_ = 3,239.87, *W* = AIC_c_ weight, *k* = number of parameters. Models comprising the top 50% cumulative *W* are shown.

Model	ΔAICc	*W*	*k*
Number of spots, 1st season	0	0.048	33
Aspect ratio, 1st season	0.44	0.039	33
Roundness^2^, 1st 3 years	0.82	0.032	34
Angle^2^, 1st season	0.87	0.031	34
Roundness, 1st season	0.95	0.030	33
Solidity, 1st season	1.06	0.029	33
Area^2^, 1st season	1.11	0.028	34
Circularity, 1st season	1.15	0.027	33
Angle^2^, 1st 3 years	1.21	0.026	34
Null model, no covariate	1.22	0.026	32
Maximum caliper, 1st season	1.30	0.025	33
PCA dimension 1, 1st year	1.63	0.021	33
Angle, 1st 3 years	1.75	0.020	33
Solidity^2^, 1st season	1.76	0.020	34
Perimeter, 1st season	1.88	0.019	33
Feret angle^2^, 1st season	1.88	0.019	34
PCA dimension 2^2^, 1st year	1.90	0.019	34
Feret angle, 1st season	1.93	0.018	33
Number of spots^2^, 1st season	2.06	0.017	34

## Discussion

We were able to objectively and reliably quantify coat pattern traits of wild giraffes using image analysis software. We demonstrated that some giraffe coat pattern traits of spot shape appeared to be heritable from mother to calf, and that coat pattern phenotypes defined by spot size and shape differed in fitness as measured by neonatal survival. Individual covariates of spot size and shape significantly affected survival during the first 4 months of life. These results support the hypothesis that giraffe spot patterns are heritable (Dagg, 1968), and affect neonatal calf survival (Langman, 1977; Mitchell & Skinner, 2003). The fact that spot patterns affected survival could be related to camouflage, but could also reflect pleiotropy of spot traits with other traits affecting fitness (Wilson & Nussey, 2010; Lailvaux & Kasumovic, 2011), or some other effect such as shared environment (Falconer & Mackay, 1996). Our methods and results add to the toolbox for objective quantification of complex mammalian coat pattern traits, and should be useful for taxonomic or phenotypic classifications based on photographic coat pattern data.

Our analyses highlighted a few aspects of giraffe spots that were most likely to be heritable and which seem to have the greatest adaptive significance. Circularity and solidity, both descriptors of spot shape, showed the highest mother-offspring similarity. Circularity describes how close the spot is to a perfect circle, and is positively correlated with the trait of roundness and negatively correlated with aspect ratio. Solidity describes how smooth and entire the spot edges are versus tortuous, ruffled, lobed, or incised and is negatively correlated with the trait of perimeter. We did not document significant mother-offspring similarity of any size-related spot traits (number of spots, area, perimeter, and maximum caliper), but the first dimension of the PCA was largely composed of size-related traits. These characteristics could form the basis for quantifying spot patterns of giraffes across Africa, and gives field workers studying any animal with complex color patterns a new quantitative lexicon for describing spots. However, our mode shade measurement was a crude metric, and color is greatly affected by lighting conditions, so we suggest standardization of photographic methods to control for lighting if color is to be analyzed in future studies.

We found that both size and shape of spots was relevant to fitness measured as juvenile survival. We observed the highest calf survival in the phenotypic group generally described as large spots that were either circular or irregular. Lowest survival was in the groups with small and medium-sized circular spots, and small irregular spots. Both the survival by phenotype analysis and the individual covariate survival analysis found that larger spots (smaller number of spots) and irregularly shaped or less-elliptical spots (smaller aspect ratio) were correlated with increased survival. It seems possible that these traits enhance the background-matching of giraffe calves in the vegetation of our study area (Ruxton, Sherratt & Speed, 2004; Merilaita, Scott-Samuel & Cuthill, 2017), and that neonatal camouflage could be an adaptive feature of complex coat patterns in other taxa (Allen et al., 2011). However, covariation in spot patterns and survival could also reflect a maternal effect, or some environmental effect. The relationships among spot traits and their effects on fitness are not well studied, and we are aware of no other study that measured coat pattern traits and related variation in those traits to fitness. Additional investigations into adaptive function and genetic architecture across many taxa are needed to fill this knowledge gap.

Whether or not spot traits affect juvenile survival via anti-predation camouflage, spot traits may serve other adaptive functions such as thermoregulation (Skinner & Smithers, 1990), or social communication (VanderWaal et al., 2014), and thus may demonstrate associations with other components of fitness, such as survivorship in older age classes or fecundity. Individual recognition, kin recognition, and inbreeding avoidance also could play a role in the evolution of spot patterns in giraffes and other species with complex coat patterns (Beecher, 1982; Tibbetts & Dale, 2007; Sherman, Reeve & Pfennig, 1997). Different aspects of spot traits may also be nonadaptive and serve no function, or spot patterns could be affected by pleiotropic selection on a gene that influences multiple traits (Lamoreux et al., 2010).

Photogrammetry to remotely measure animal traits has utilized geometric approaches that estimate trait sizes using laser range finders and known focal lengths (Lyon, 1994; Lee et al., 2016a), photographs of the traits together with a predetermined measurement unit (Ireland et al., 2006; Willisch, Marreros & Neuhaus, 2013), or lasers to project equidistant points on animals while they are photographed (Bergeron, 2007). We hope the framework we have described using ImageJ software to quantify spot characteristics with trait measurements from photographs will prove useful to future efforts at quantifying animal markings as in animal biometry (Kühl & Burghardt, 2013). Trait measurements and cluster analysis such as we performed here could also be useful to classify subspecies, phenotypes, or other groups based on variation in markings, which could advance the field of phenomics for organisms with complex skin or coat patterns (Houle, Govindaraju & Omholt, 2010).

Patterned coats of mammals are hypothesized to be formed by two distinct processes: a spatially oriented developmental mechanism that creates a species-specific pattern of skin cell differentiation and a pigmentation-oriented mechanism that uses information from the pre-established spatial pattern to regulate the synthesis of melanin (Eizirik et al., 2010). The giraffe skin has more extensive pigmentation and wider distribution of melanocytes than most other animals (Dimond & Montagna, 1976). Coat pattern variation may reflect discrete polymorphisms potentially related to life-history strategies, a continuous signal related to maternal effects, or a combination of both. Future work on the genetics of coat patterns will hopefully shed light upon the mechanisms and consequences of coat pattern  variation.

## Conclusions

Our evidence that coat pattern traits were related to juvenile survival is an important finding that adds an incremental step to our understanding of the evolution of animal coat patterns. We expect the application of image analysis to giraffe coat patterns will also provide a new, robust dataset to address taxonomic and evolutionary hypotheses. For example, two recent genetic analyses of giraffe taxonomy both placed Masai giraffes as their own species (Brown et al., 2007; Fennessy et al., 2016), but the lack of quantitative tools to objectively analyze coat patterns for taxonomic classification may underlie some of the confusion that currently exists in giraffe systematics (Bercovitch et al., 2017).

##  Supplemental Information

10.7717/peerj.5690/supp-1 Table S1 Summary statistics of variance explained by dimensions of a principal components analysis (PCA) of 11 giraffe spot trait variables

10.7717/peerj.5690/supp-2 Table S2 Pearson correlation coefficients among giraffe spot trait variables Bold correlation coefficients are statistically significant at alpha = 0.05.

10.7717/peerj.5690/supp-3 Figure S1 Spatial distribution of the four phenotype groups of giraffe calves defined by k-means clustering of multivariate spot traits Mixed spatial distribution of calves shows no clustering by phenotype which could contribute to a shared environment effect. Mixed spatial distribution also shows permanent emigration should be random in relation to spot phenotypes.

10.7717/peerj.5690/supp-4 Figure S2 Residual plots for PO regressions of spot traits Diagnostic plots generated by qqplot.

10.7717/peerj.5690/supp-5 Figure S3 Representative spot outlines from Masai giraffes in northern Tanzania and their corresponding circularity and solidity values Ranges of spot trait values from 258 calves are given in parentheses.

10.7717/peerj.5690/supp-6 Figure S4 Contribution of spot trait variables to principal components analysis first and second dimensions Percent contributions of each spot trait variable to the first two dimensions. P, perimeter, MC, maximum caliper, N , number of spots, C, circularity, AR, aspect ratio, R, roundness, S, solidity, FA, Feret angle.

## Additional Information and Declarations

### Field Study Permissions

The following information was supplied relating to field study approvals (i.e., approving body and any reference numbers): This research was carried out with permission from the Tanzania Commission for Science and Technology (COSTECH), Tanzania National Parks (TANAPA), the Tanzania Wildlife Research Institute (TAWIRI). COSTECH research permit numbers: 2017-163-ER-90-172, 2016-146-ER-2001-31, 2015-22-ER-90-172, 2014-53-ER-90-172, 2013-103-ER-90-172, 2012-175-ER-90-172, 2011-106-NA-90-172.

### Data Availability

The following information was supplied regarding data availability: Lee D, Cavener DR, Bond M. Data from: Seeing spots: Measuring, quantifying heritability, and assessing fitness consequences of coat pattern traits in a wild population of giraffes (Giraffa camelopardalis). Dryad Digital Repository. https://doi.org/10.5061/dryad.6514r.
