# Supplementary material for: Seeing spots: quantifying mother-offspring similarity and assessing fitness consequences of coat pattern traits in a wild population of giraffes (Giraffa camelopardalis)
Source: PeerJ. 2018 Oct 2;6:e5690. doi: 10.7717/peerj.5690 (PMC6173159; doi:10.7717/peerj.5690)
Supplement: Table S1 [file peerj-06-5690-s001.docx]

**Supplementary Material Table S1**. Summary statistics of variance explained by dimensions of a principal components analysis (PCA) of 11 giraffe spot trait variables.

| PCA dimension | Eigenvalue | % variance explained | Cumulative % variance |
| --- | --- | --- | --- |
| 1 | 3.2 | 40.5 | 40.5 |
| 2 | 1.9 | 24.0 | 64.5 |
| 3 | 1.6 | 19.2 | 83.7 |
| 4 | 0.7 | 9.1 | 92.8 |
| 5 | 0.3 | 3.3 | 96.1 |
| 6 | 0.2 | 2.2 | 98.3 |
| 7 | 0.1 | 1.4 | 99.7 |
| 8 | 0.0 | 0.3 | 100.0 |
