# Supplementary material for: Seeing spots: quantifying mother-offspring similarity and assessing fitness consequences of coat pattern traits in a wild population of giraffes (Giraffa camelopardalis)
Source: PeerJ. 2018 Oct 2;6:e5690. doi: 10.7717/peerj.5690 (PMC6173159; doi:10.7717/peerj.5690)
Supplement: Table S2 — Bold correlation coefficients are statistically significant at alpha = 0.05. [file peerj-06-5690-s002.docx]

|  | Number | Area | Perimeter | Angle | Circularity | Maximum Caliper | Feret Angle | Aspect Ratio | Roundness | Solidity | PCA 1^st^ Dimension | PCA 2^nd^ Dimension |
| --- | --- | --- | --- | --- | --- | --- | --- | --- | --- | --- | --- | --- |
| Number | 1 | **-0.82** | **-0.81** | -0.01 | **0.42** | **-0.84** | -0.09 | 0.23 | -0.20 | **0.27** | **-0.86** | -0.17 |
| Area | **-0.82** | 1 | **0.90** | 0.07 | **-0.30** | **0.95** | 0.08 | **-0.30** | **0.25** | -0.04 | **0.82** | **0.26** |
| Perimeter | **-0.81** | **0.90** | 1 | 0.03 | **-0.64** | **0.95** | 0.05 | -0.24 | 0.17 | **-0.39** | **0.87** | 0.06 |
| Angle | -0.01 | 0.07 | 0.03 | 1 | 0.06 | 0.06 | **0.78** | -0.03 | 0.01 | 0.11 | -0.16 | **0.28** |
| Circularity | **0.42** | **-0.30** | **-0.64** | 0.06 | 1 | **-0.50** | 0.04 | -0.22 | **0.27** | **0.86** | **-0.56** | **0.46** |
| Maximum Caliper | **-0.84** | **0.95** | **0.95** | 0.06 | **-0.50** | 1 | 0.06 | -0.19 | 0.13 | -0.25 | **0.87** | 0.13 |
| Feret Angle | -0.09 | 0.08 | 0.05 | **0.78** | 0.04 | 0.06 | 1 | -0.10 | 0.05 | 0.14 | -0.10 | **0.31** |
| Aspect Ratio | 0.23 | -0.30 | -0.24 | -0.03 | -0.22 | -0.19 | -0.10 | 1 | **-0.94** | **-0.35** | -0.10 | **-0.59** |
| Roundness | -0.20 | **0.25** | 0.17 | 0.01 | **0.27** | 0.13 | 0.05 | **-0.94** | 1 | **0.36** | 0.07 | **0.61** |
| Solidity | **0.27** | -0.04 | **-0.39** | 0.11 | **0.86** | -0.25 | 0.14 | **-0.35** | 0.36 | 1 | **-0.38** | **0.56** |
| PCA 1^st^ Dim | **-0.86** | **0.82** | **0.87** | -0.16 | **-0.56** | **0.87** | -0.10 | -0.10 | 0.07 | **-0.38** | 1 | 0 |
| PCA 2^nd^ Dim | -0.17 | **0.26** | 0.06 | **0.28** | 0.46 | 0.13 | **0.31** | **-0.59** | **0.61** | **0.56** | 0 | 1 |
