# Supplementary figures and images for: Seeing spots: quantifying mother-offspring similarity and assessing fitness consequences of coat pattern traits in a wild population of giraffes (Giraffa camelopardalis)
Source: PeerJ. 2018 Oct 2;6:e5690. doi: 10.7717/peerj.5690 (PMC6173159; doi:10.7717/peerj.5690)

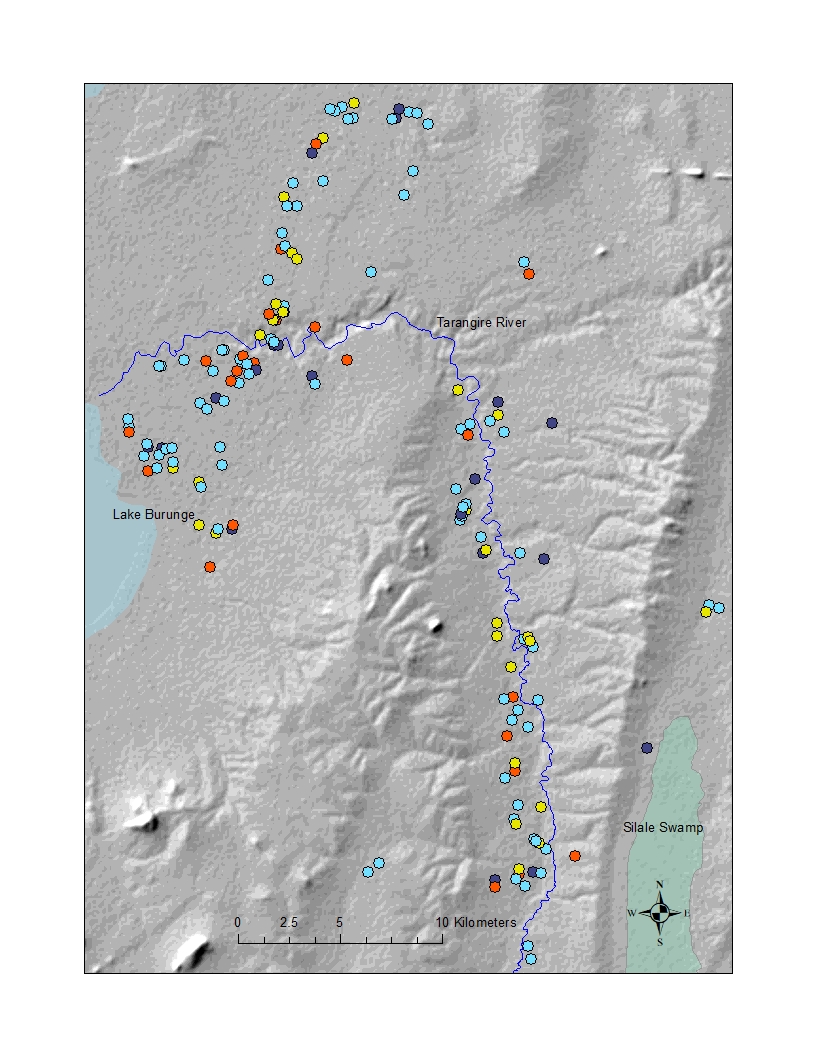

Supplement: Figure S1 — Mixed spatial distribution of calves shows no clustering by phenotype which could contribute to a shared environment effect. Mixed spatial distribution also shows permanent emigration should be random in relation to spot phenotypes. [file peerj-06-5690-s003.jpg]

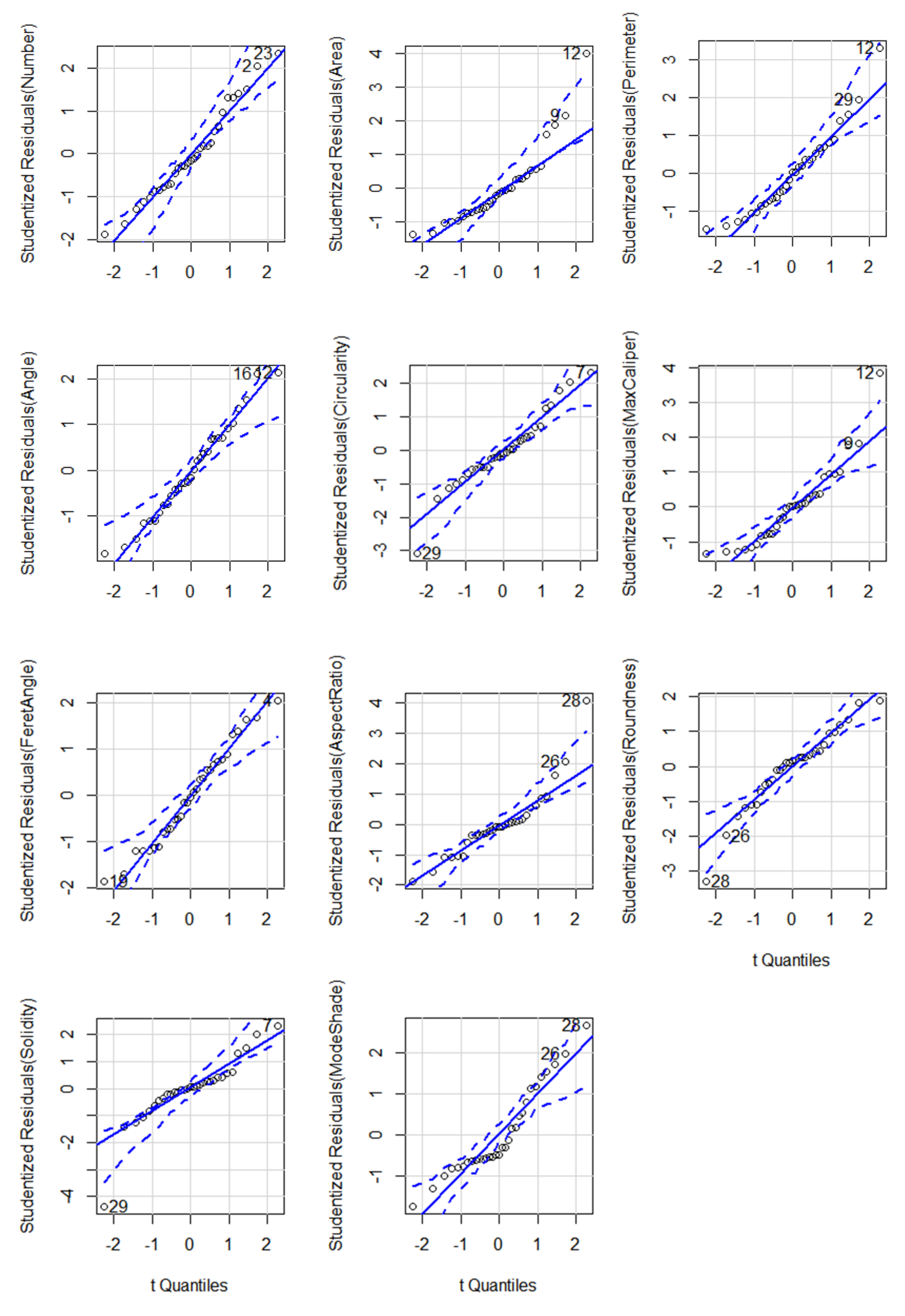

Supplement: Figure S2 — Diagnostic plots generated by qqplot. [file peerj-06-5690-s004.png]

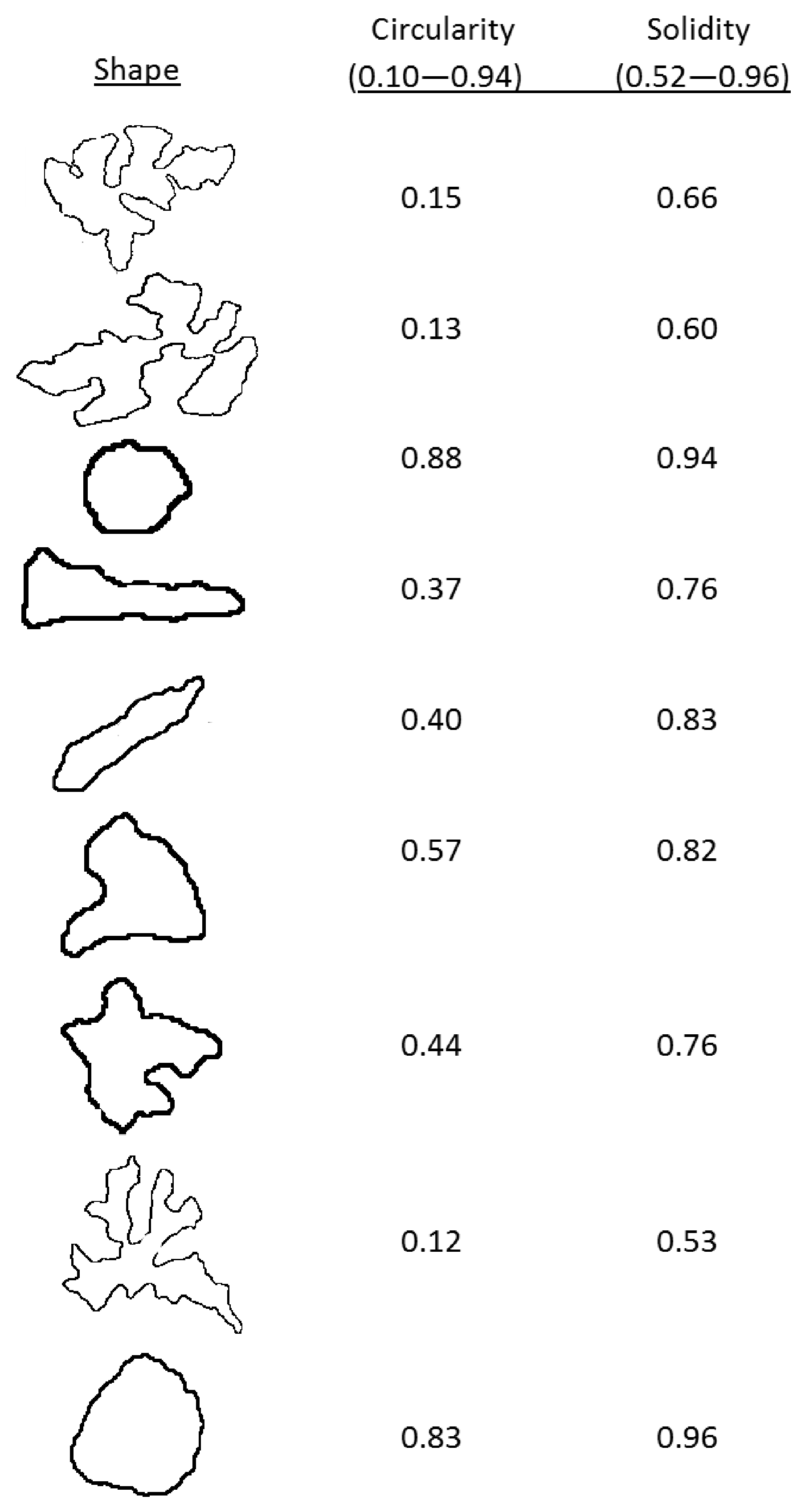

Supplement: Figure S3 — Ranges of spot trait values from 258 calves are given in parentheses. [file peerj-06-5690-s005.png]

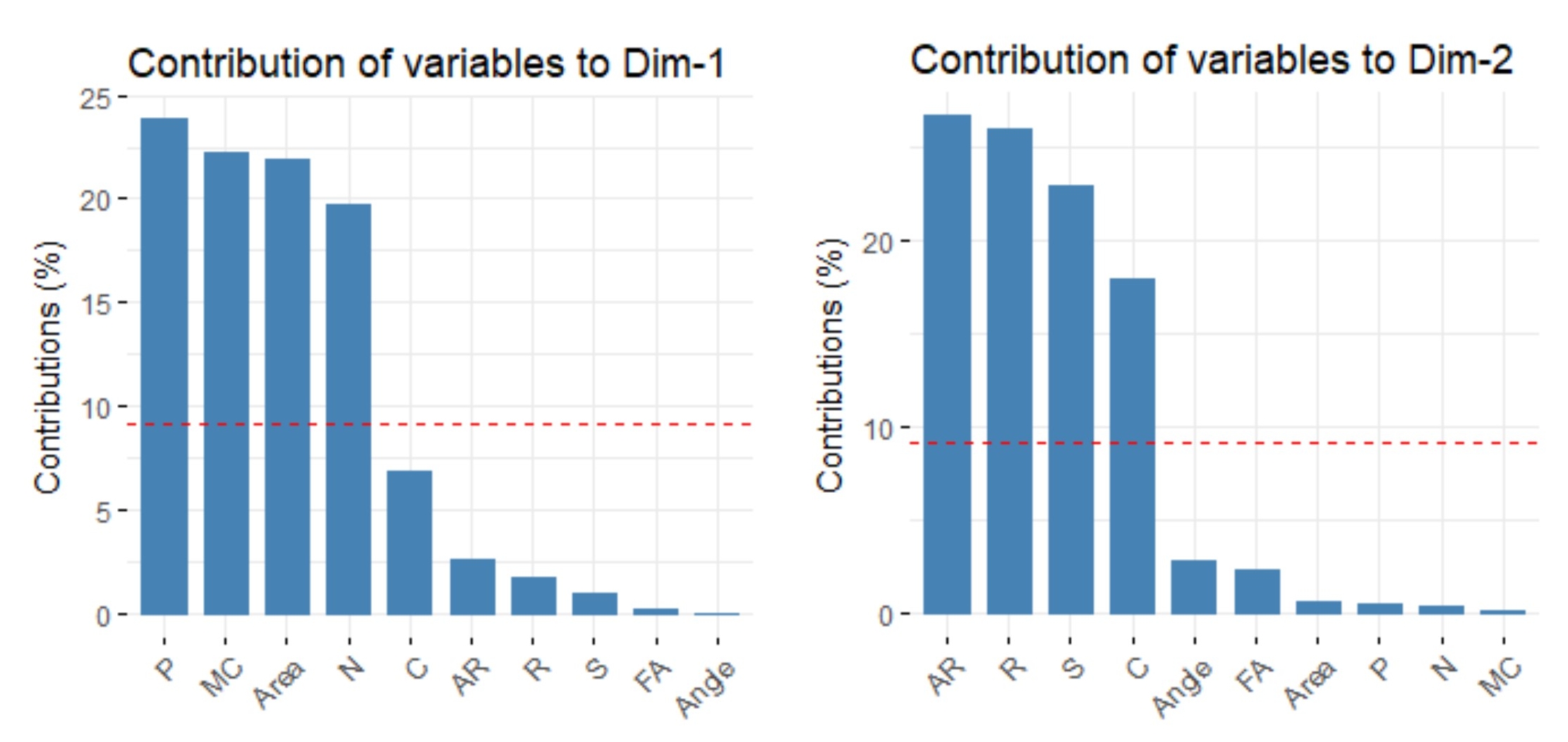

Supplement: Figure S4 — Percent contributions of each spot trait variable to the first two dimensions. P, perimeter, MC, maximum caliper, N , number of spots, C, circularity, AR, aspect ratio, R, roundness, S, solidity, FA, Feret angle. [file peerj-06-5690-s006.jpg]
